# Determining the Relative Contribution and Hierarchy of *hha* and *qseBC* in the Regulation of Flagellar Motility of *Escherichia coli* O157:H7

**DOI:** 10.1371/journal.pone.0085866

**Published:** 2014-01-21

**Authors:** Vijay K. Sharma, Thomas A. Casey

**Affiliations:** Food Safety and Enteric Pathogens Research Unit, United States Department of Agriculture, Agricultural Research Service, National Animal Disease Center, Ames, Iowa, United States of America; U. S. Salinity Lab, United States of America

## Abstract

In recent studies, we demonstrated that a deletion of *hha* caused increased secretion of locus of enterocyte encoded adherence proteins and reduced motility of enterohemorrhagic *Escherichia coli* (EHEC) O157:H7. In addition to the importance of *hha* in positive regulation of motility, a two-component quorum sensing pathway encoded by the *qseBC* genes has been shown to activate bacterial motility in response to mammalian stress hormones epinephrine and norepinephrine as well as bacterially produced autoinducer-3. In this study, we compared regulatory contribution and hierarchy of *hha*, a member of the Hha/YmoA family of nucleoid-associated proteins, to that of *qseBC* in the expression of EHEC O157:H7 motility. Since norepinephrine affects motility of EHEC O157:H7 through a *qseBC*-encoded two-component quorum sensing signaling, we also determined whether the *hha*-mediated regulation of motility is affected by norepinephrine and whether this effect is *qseBC* dependent. We used single (Δ*hha* or Δ*qseC*) and double (Δ*hha* Δ*qseC*) deletion mutants to show that *hha* exerts a greater positive regulatory effect in comparison to *qseBC* on the expression of motility by EHEC O157:H7. We also show that Hha is hierarchically superior in transcriptional regulation of motility than QseBC because transcription of *qseC* was significantly reduced in the *hha* deletion mutant compared to that in the parental and the *hha*-complemented mutant strains. These results suggest that *hha* regulates motility of EHEC O157:H7 directly as well as indirectly by controlling the transcription of *qseBC*.

## Introduction

Enterohemorrhagic (EHEC) *Escherichia coli* O157:H7 causes a broad spectrum of diarrheal illnesses, including uncomplicated diarrhea, hemorrhagic colitis, and hemolytic uremic syndrome [1]. Cattle are the major reservoir for EHEC O157:H7, which colonizes the terminal portion called the recto-anal junction or RAJ of the large intestine of these animals [2], [3]. The locus of enterocyte effacement (LEE) [4] encodes a type III secretion system for secreting various LEE and non-LEE-encoded proteins [5] that are required for the colonization of cattle intestines and for the formation of characteristic histopathology, termed attaching and effacing lesions [6], on intestinal tissues [7]–[9]. EHEC O157:H7 colonization of cattle intestines leads to increased fecal shedding of these bacteria, a major risk factor in the contamination of beef and other bovine food products [10].

Motility is essential for pathogenicity of many bacterial pathogens [11], [12], and most EHEC O157:H7 strains associated with large disease outbreaks in humans have been shown to express flagella and tend to be motile [13], [14]. However, the role of flagellar motility in EHEC O157:H7 colonization of bovine intestines and human infections had remained ambiguous. Bovine experimental infection studies have demonstrated that flagella are dispensable for the EHEC O157:H7 colonization in these animals [15]. That motility and/or flagella might not be required for human virulence is suggested by the increased isolation of sorbitol-fermenting non-motile EHEC O157:NM strains from HUS patients in Germany that adhered at significantly higher levels to human colonic epithelial cells and expressed increased amounts of curli [16]. However, in a recent study we have demonstrated that a *hha* deletion mutant of EHEC O157:H7 expressing LEE at very high levels but showing reduced motility due to the reduced expression of the flagellar gene *fliC* failed to establish increased colonization of cattle intestines compared to the wild-type strain [17]. Despite unsettled role of motility in EHEC O157:H7 colonization of bovine intestines, there is increasing evidence that flagella might promote adherence of EHEC O157:H7 to the target sites in the large intestine. For example, it has recently been shown that a *fliC* mutant of EHEC O157:H7 adhered poorly to the cultured primary rectal epithelial cells compared to the *fliC* complemented mutant strain [18]. In addition, these and other studies have also demonstrated that the flagellar expression is temporally regulated as EHEC O157:H7 bacterial cells show abundant flagella on their cell surfaces during the early stages of adherence to the epithelial cells but the flagellar expression is reduced during the formation of attaching and effacing lesions on these cells [13], [18]. Many enteropathogenic *E. coli* (EPEC) serotypes have also been shown to require flagella for adherence and formation of microcolonies on HeLa or HEp-2 cells [19], but unlike H7 flagella, purified flagella of EPEC serotypes failed to adhere to the rectal epithelial cells [18], implying a specificity of H7 flagella for the rectal epithelial cells.

Transcriptional regulation of LEE and flagellar genes conferring phenotypes of intimate adherence and motility, respectively, is highly complex. Several transcriptional regulators control the expression of these two important sets of genes in response to complex networks of environmental and physiological cues [20], [21]. For example, the expression of LEE, which consists of five major operons named *LEE1* – *LEE5*, is positively regulated by the master regulator Ler encoded by the *ler* gene of *LEE1* [22]. Transcriptional factors, such as IHF, QseA, GrlA, LrhA, and Pch activate *ler* transcription [23]–[27] while others, such as H-NS, Hha, Hfq, and SdiA, repress transcription of *ler* [25], [28]–[33]. Transcriptional regulation of flagellar genes is governed by the master regulator FlhDC complex [34], whose expression is positively modulated by H-NS, Hha, CsrA, OmpR, cAMP-CAP, and QseBC [14], [35]–[38] and negatively by SdiA, GrlA, LrhA, and HdfR [13], [29], [39], [40].

In previous reports, we have demonstrated that a hemolysin modulating protein Hha exerts a negative effect on LEE expression by repressing the transcription of *ler* and a positive effect on flagellar gene expression by activating *flhDC* transcription [32], [35]. Similarly, the QseBC encoded quorum sensing system, like Hha, has been shown to exert positive regulatory effects on flagellar gene expression by the activation of *flhDC* through direct interactions of QseB with the *flhDC* promoter [14]. In the QseBC system, QseC is a transmembrane histidine sensor kinase that autophosphorylates and transfers the phosphoryl moiety to its cognate response regulator QseB, which interacts with the regulatory sequences of its target genes, such as *flhDC*, to affect their expression [14], [41]. QseBC also enhances LEE expression through a quorum sensing regulator QseA, which is activated indirectly by QseBC through the activation of quorum sensing *qseEF* genes in response to bacterially synthesized autoinducers and mammalian stress hormones epinephrine or norepinephrine [42].

Although both Hha and QseBC affect flagellar gene expression by increasing the transcription of the master regulator FlhDC [14], [35], [43], neither the relative contribution nor the hierarchy of these two genetic systems in the regulation of *flhDC* and bacterial motility is fully understood. In this study, we used single gene deletion mutants, lacking either the *hha* (Δ*hha*) or the *qseC* (Δ*qseC*) gene, to compare the magnitude of the regulatory effects of Hha to that of the well-characterized positive effects of QseBC on bacterial motility [14] in the presence or absence of norepinephrine. We selected the *qseC* deletion mutant for this comparison since the *qseC* mutant has previously been shown to exhibit reduced motility due to its inability to respond to norepinephrine [14], [44]. In addition, we used a double deletion mutant, lacking *hha* and *qseC* (Δ*hha* Δ*qseC*), to confirm both the magnitude of contribution and hierarchy of Hha relative to QseBC in the complex regulatory network controlling motility of EHEC O157:H7.

## Materials and Methods

### Bacterial strains, culture media, and growth conditions

Bacterial strains and plasmids used in this study are listed in Table 1. A *stx2* deletion mutant strain (NADC 6431) [32] derived from a streptomycin-resistant mutant of enterohemorrhagic *Escherichia coli* (EHEC) O157:H7 strain 86–24 [45], [46] was used as a parent strain for constructing the isogenic *hha* (NADC 6491), *qseC* (NADC 6488), and *hha qseC* (NADC 6528) deletion mutants (Table 1). Bacterial strains were cultivated in Luria-Bertani broth (LB) (Sigma-Aldrich, St. Louis, MO) or LB agar supplemented with antibiotics (Sigma-Aldrich) as needed (streptomycin, 100 mg liter^−1^; kanamycin 50 mg liter^−1^; and carbenicillin 100 mg liter^−1^).

**Table 1:** Bacterial strains and plasmids^a^

Strain or plasmid	Genotype and description	Source or reference
***E. coli*** ** strains**		
NADC 5570	*stx2^+^* and streptomycin-resistant *E. coli* O157:H7	[45], [46]
	strain 86-24	
NADC 6431	Δ*stx2* derivative of NADC 5570	[32]
NADC 6491	Δ*hha* mutant strain of NADC 6431	[32]
NADC 6488	Δ*qseC* mutant strain of NADC 6431	This study
NADC 6528	Δ*qseC* mutant strain of NADC 6491	This study
TOP 10	F^-^ *mcrA* Δ (*mrr-hsd*RMS-*mcr*BC) Φ80*lacZ*ΔM15	Life Technologies
	Δ*lac*X74 *rec*A1 *ara*D139 Δ (*ara*-*leu*)7697 *gal*U	
	*gal*K *rpsL* (Str^R^) *end*A1 *nup*G	
**Plasmids**		
pSMART-LC	Cloning vector	Lucigen
pCRXL and pCR2.1	Cloning vectors	Life Technologies
pAM450	Plasmid with a temperature-sensitive origin of	[32], [46]
	replication	
pSM400	pCRXL containing a 3.0 kb US-DS fragment for	This study
	deleting the *qseC* gene	
pSM488	*qseC* deletion plasmid constructed by cloning a	This study
	3.0 kb US-DS fragment of pSM400 at the *Xba*I	
	site of pAM450	
pSM639	pSMART-LC containing 3.51 kb fragment for	This study
	complementing the *qseC* deletion mutant	
pSM197R	pCR2.1 carrying a 630 bp DNA fragment	[32], [56]
	encoding *hha*	

a Detailed descriptions of the construction of bacterial strains and plasmids listed in this table are provided under material and methods section.

### Recombinant DNA procedures

For constructing an in-frame *qseC* deletion mutant of EHEC O157:H7 strain NADC 6431, a 1.5 kb DNA fragment upstream (US) and a 1.5 kb DNA fragment downstream (DS) of the *qseC* ORF of this strain were isolated by PCR using primer pairs *qseC*-US_F_/*qseC*-US_R_ and *qseC*-DS_F_/*qseC*-DS_R_, respectively (Table 2). The *Xba*I restriction sites were built into primers *qseC*-US_F_ and *qseC*-DS_R_ and *Bgl*II restriction sites were built into primers *qseC*-US_R_ and *qseC*-DS_F_. The US and DS fragments were ligated together in the 5′US-DS3′ order at the *Bgl*II site located at the 3′ and 5′ ends of the US and DS fragments, respectively. The 3.0 kb US-DS fragment was cloned into pCRXL and electroporated into *E. coli* TOP10 bacterial cells according to the manufacturer's instructions (Life Technologies, Grand Island, NY) to generate a recombinant plasmid pSM400 (Table 1). The pSM400 plasmid DNA was digested with *Xba*I, digested DNA subjected to standard agarose gel electrophoresis, and 3.0 kb US-DS *Xba*I fragment was extracted from an agarose gel using a Gel Extraction Kit according to the manufacturer's instructions (Qiagen, Valencia, CA). The 3.0 kb US-DS *Xba*I fragment was cloned at the *Xba*I site of a plasmid (pAM450) temperature-sensitive for its replication [32], [46]. The recombinant of pAM450 carrying the 3.0 kb *Xba*I fragment (pSM488) (Table 1) was electroporated into the parent strain (NADC 6431) for deleting the *qseC* gene by using a previously described method [32]. The resulting *qseC* deletion mutant was named NADC 6488 (Table 1). The *hha* mutant (NADC 6491) described in a previous study [32] was used as a host strain for the plasmid pSM488 that allowed deletion of the gene *qseC* to generate a *hha qseC* double deletion mutant (NADC 6528) (Table 1) by using the same procedure as was used for deleting the *qseC* gene. The deletion of the *qseC* gene in the parental and the *hha* mutant strains was confirmed by PCR using the *qseC* deletion primers listed in Table 2. A 3.51 kb DNA fragment containing the *qseB* and *qseC* genes was isolated from EHEC O157:H7 strain 5570 by PCR using the primers *qseBC-*US_F_ and *qseBC-*DS_R_ and cloned into a low-copy vector pSMART-LC (Lucigen Corporation, Middleton, WI). The recombinant *qseBC*-pSMART-LC (pSM639) was used for *in trans* complementation of the *qseC* and *hha qseC* deletion mutants. We used plasmid pSM197R (pCR2.1 carrying *hha*), whose construction has been described in a previous report [32], for *in trans* complementation of the *hha* and *hha qseC* deletion mutants for *hha*. Empty vectors pSMART-LC and pCR2.1 were electroporated into the parent strain NADC 6431 to construct strains NADC 6431/pSMART-LC and NADC 6431/pCR2.1 that were used as controls (Table 1).

**Table 2:** Primers used for PCR and QRT-PCR.

Primer	Nucleotide sequence^a^	Location^b^
***E. coli*** ** Primers** c		
*qseC*-US_F_	GATTCTAGAGGCTTTGGTTAACAGGAGAAAG	3975818–3975839
*qseC*-US_R_	GAAAGATCTAGATCTGGTACGAATAAAAT	3977194–3977173
	CACTACCG	
*qseC*-DS_F_	GCGAGATCTAGATCTAGGGTAAGACTTTTG	3978576–3978598
	CTAAATTC	
*qseC*-DS_R_	GACTCTAGACACGACTATTCAACCGCATAG	3980066–3980046
*qseBC*-_F_	GATTCTAGAGGCTTTGGTTAACAGGAGAAAG	3975818–3975839
*qseBC*-_R_	GATCTCTAGAGAGTACGGATTTCTGCGATTAC	3979328–3979297
*qseC*_F_ (*qseC* deletion	CTGGCGAACCCTTAACACTG	3977001–3977020
confirmation primer)		
*qseC*_R_ (*qseC* deletion	TCGTTCAGTTGACCATTGGAG	3978750–3978729
confirmation primer)		
*qseC*_F_ (QRT-PCR)	CTGGCGAACCCTTAACACTG	3977507–3977526
*qseC*_R_ (QRT-PCR)	TCCAGACAAAACGCCATTGA	3977626–3977607
*qseC*_p_ (QRT-PCR)	FAM/TGGCGATAACGGAGAAGATATTCC/TAM	3977529–3977552
*rpoA*_F_ (QRT-PCR)	GGCTTGACGATTTCGACATC	4242887–4242906
*rpoA*_R_ (QRT-PCR)	GGTGAGAGTTCAGGGCAAAG	4242997–4242978
*rpoA*_p_ (QRT-PCR)	TGAAGTTATTCTTACCTTGAATAAATC	4242976–4242942

a Nucleotide sequences of primers used in this study were selected from the published genome sequence of *E. coli* O157:H7 strain EDL933 [57] with the accession number AE005174.2.

b Location refers to the position of primer sequence in the genome of EDL933 [57].

c Subscripts F, R, and P denotes forward primer, reverse primer, and TaqMan probe; (QRT-PCR) denotes quantitative reverse transcriptase-based PCR.

### Quantitative reverse transcription PCR (QRT-PCR)

Bacterial strains were grown for five h (A_600_ of 1.1 to 1.2) in low-glucose DMEM (Life Technologies). One ml aliquots of these bacterial cultures were mixed with two ml of RNA-Protect solution and the total RNA was isolated by using the RNeasy Kit according to the manufacturer's instructions (Qiagen). RNA was subjected to DNase treatment by using the Ambion TURBO DNA-free Kit to remove DNA contamination according to the manufacturer's instructions (Life Technologies). QRT-PCR was performed by adding 25 ng of DNase-treated RNA, 0.75 µM each of antisense and sense primers, 0.25 µM of a TaqMan probe (labeled at the 5′ end with FAM reporter and at 3′ end with TAMRA quencher) (Integrated DNA Technologies, Coralville, IA), to a QRT-PCR Master Mix (Agilent Technologies, Santa Clara, CA). Primers and labeled probes used for QRT-PCR are listed in Table 2. QRT-PCR was carried out in Mx3005P qPCR System (Agilent) by using the following parameters: 50°C for 30 min for cDNA synthesis; 95°C for 10 min; 35 cycles of 95°C for 30 sec, 55°C for 60 sec, and 72°C for 30 sec for cDNA amplification and detection of amplification products. The fold-change in the gene expression was plotted by adjusting the expression of a target gene to one for the parent (calibrator) strain and by normalizing to the expression of the house-keeping gene *rpoA*.

### Determination of bacterial motility

Bacterial strains were grown in LB broth containing-100 µg ml^−1^ of carbenicillin at 37°C with shaking (175 rpm) for 18–24 h. These cultures were standardized to OD_600_ of 4.00 and 2 µl from each culture was spotted on soft-agar motility plates (DMEM containing 0.3% Noble agar, 100 µg ml^−1^ carbenicillin and with or without 50 µM norepinephrine). The diameters of motility halos produced around the point of inoculations were measured after incubating motility plates for 18–24 h at 37°C.

### Statistical analyses

A two sample, nonparametric Mann-Whitney test was used to determine the significance of the differences in motilities and expression of selected genes in the mutant and the complemented mutants relative to the parental strain. The differences were considered significant at *p*<0.05.

## Results

### Hha affects motility by a greater magnitude than QseBC

**Figure 1:**
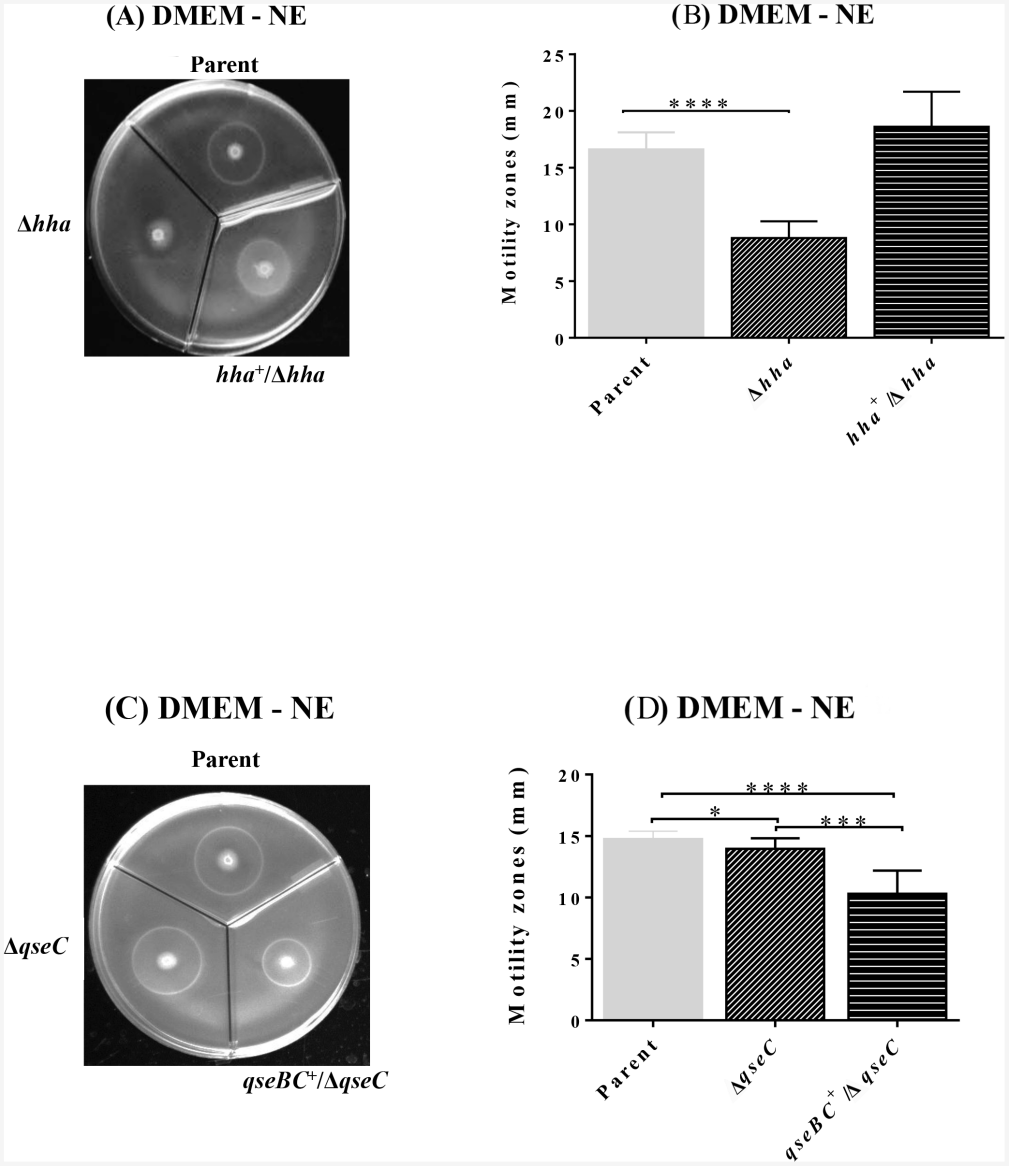
Relative effects of the *hha* and *qseC* deletions on bacterial motility. Three biological and a total of nine technical replicates of the overnight bacterial cultures of each strain were standardized to equivalent optical densities (*A*_600 nm_ = 4.00) and spotted in 2 µl aliquots on the motility agar plates containing or lacking norepinephrine (NE). Figures 1A and 1B show the motility zones produced around the point of bacterial inoculations and plot of their corresponding diameters (mm), respectively, for *hha* (Δ*hha*), *hha*-complemented (*hha* ^+^/Δ*hha*) mutants, and the parent strain. Figures 1C and 1D show the motility zones produced around the point of bacterial inoculations and plot of their corresponding diameters (mm), respectively, for *qseC* (Δ*qseC*), *qseC*-complemented (*qseBC* ^+^/Δ*qseC*) mutants, and the parent strain. Statistical significance of the differences in the motilities of the mutant and the complemented mutant relative to the parent strain is indicated by * (*p = *0.0177); *** (*p* = <0.003); **** (*p* = <0.0001).

Since both Hha and QseBC enhance motility of EHEC O157:H7 by the transcriptional activation of *flhDC* [14], [35], we first determined the magnitude of the effect of Hha on the expression of motility by comparing the motility of a *hha* deletion mutant to that of a *qseC* deletion mutant, which according to the published reports shows significantly reduced motility in comparison to the parent strain [14], [44]. In the present study, we observed that the motility of the *hha* deletion mutant (8.8 mm±0.47; *p* = <0.0001) was highly reduced compared to the parental (16.6 mm±0.48) (Figs. 1A and 1B) and the *qseC* deletion mutant strains (14.0 mm±0.27; *p* = 0.0177) (Figs. 1C and 1D). The complementation of the *hha* deletion mutant with a *hha-*carrying plasmid (pSM197R) restored motility of the mutant strain (18.6 mm±0.98) similar to the parental level (16.6 mm±0.48) as there was no significant (*p* = 0.1065) difference in the motilities of these two strains (Figs 1A and 1B). On the other hand, the magnitude of motility (14.0 mm±0.3) of the *qseC* deletion mutant (NADC 6488) was only slightly but significantly lower (*p* = 0.0177) than the motility (14.7 mm±0.02) of the parental strain (NADC 6431) (Figs. 1C and 1D). In addition, *in trans* complementation of the *qseC* deletion mutant with a plasmid-cloned copy of *qseBC* (pSM639) resulted in a significant reduction in motility (10.3 mm±0.6; *p* = <0.0001) compared to both the *qseC* mutant (14.0 mm±0.3) and the parental strain (14.7 mm±0.2) (Figs. 1C and 1D). This negative effect of *qseBC-*mediated complementation could presumably result from the increased expression of QseBC causing abnormal stoichiometry of non-phosphorylated to phosphorylated QseB that in turn reduces motility of the complemented *qseC* mutant in the absence of NE.

### Norepinephrine enhances motility irrespective of the presence or absence of *hha* or *qseC*

**Figure 2:**
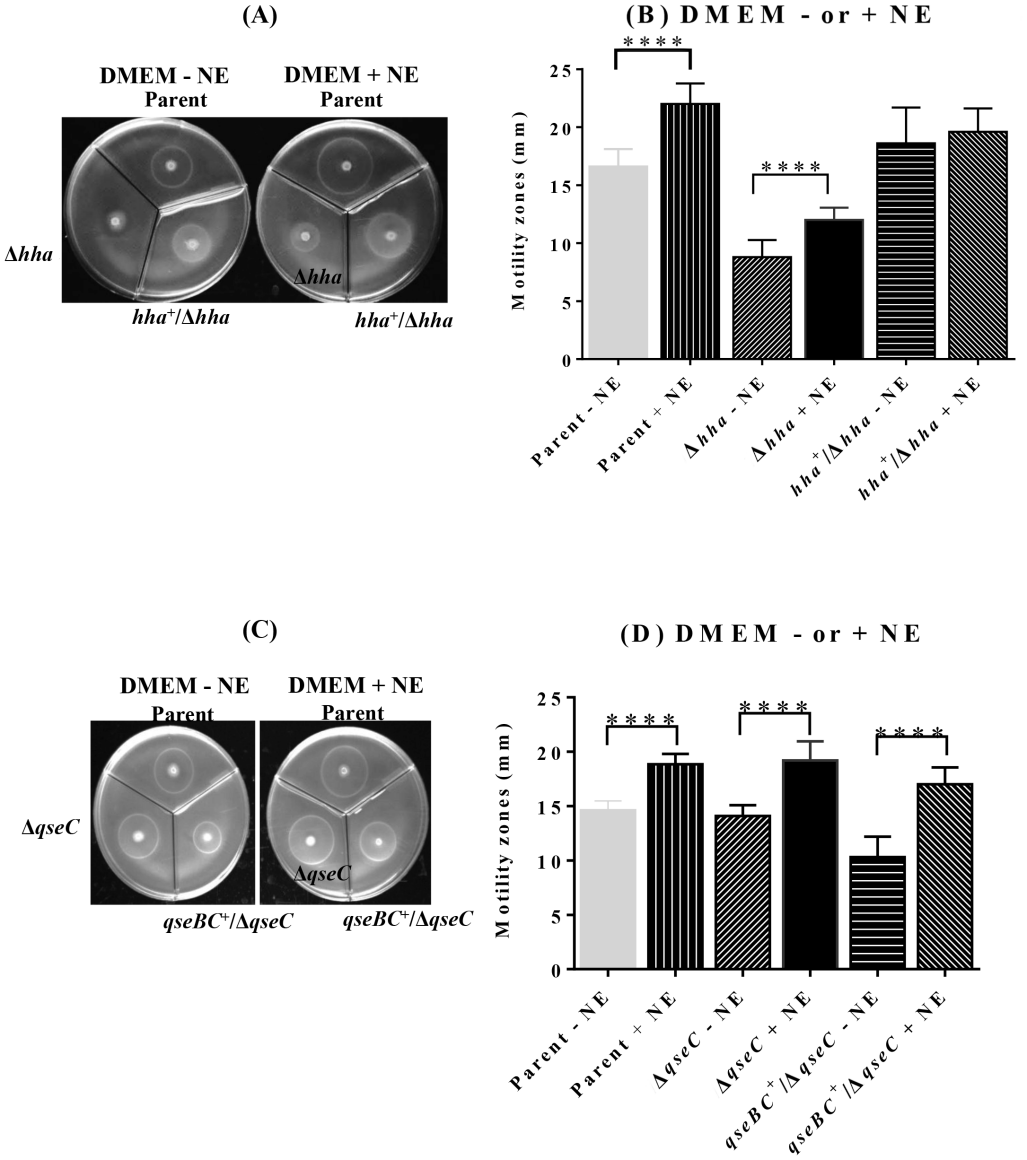
Magnitude of the motility in response to NE signaling in the *hha* and *qseC* mutants. The response to norepinephrine (NE) was determined by spotting the overnight bacterial cultures standardized to equivalent OD_600_ of 4.00 on soft-motility agar plates. The motility zones produced by each strain after an overnight of incubation at 37°C in the absence of NE were compared to those produced by the same strain in the presence of NE. Figures 2A and 2B show the motility zones produced around the point of bacterial inoculations and plot of their corresponding diameters (mm), respectively, for the parent, *hha* (Δ*hha*), and *hha*-complemented (*hha* ^+^/Δ*hha*) mutants strains in the absence or presence of NE. Figures 2C and 2D show motility zones produced around the point of bacterial inoculations and the plot of their corresponding diameters (mm), respectively, for the parent, *qseC* (Δ*qseC*), *qseC*-complemented (*qseBC* ^+^/Δ*qseC*) mutant strains in the absence or presence of NE. Statistical significance of the differences in the motilities of the mutant and the complemented mutants relative to the parental strains is indicated by **** (*p* = <0.0001).

Catecholamines, such as norepinephrine (NE) and epinephrine (E), have been shown to enhance EHEC O157:H7 motility by activation of the *qseBC*-encoded quorum sensing signaling system, and the mutants lacking *qseC* are unable to sense NE and therefore show reduced motility [47], [48]. The NE dependency of the QseBC-mediated positive regulation of motility is reflected in the absolute requirement of the phosphorylated QseB, the transcriptional factor responsible for the up-regulation of *flhDC* encoding FlhDC, the master positive regulator of motility [14], [44]. However, there is no evidence to date demonstrating any direct or indirect role of NE or QseBC in the Hha-mediated positive regulation of motility in EHEC O157:H7. A comparison of the motility of the *hha* mutant grown with or without NE revealed that although the motility of the *hha* mutant increased from 8.8 mm±0.47 without NE to 12.0 mm±0.33 (*p* = <0.0001) with NE, but it was still lowest in magnitude compared to the motility of the parental strain (16.6 mm±0.48 without NE to 22.0±0.56 with NE; *p* = <0.0001) and the *hha* mutant complemented with the *hha*-carrying plasmid pSM197R (18.6 mm±0.98 without NE to 19.6±0.64 with NE; *p* = 0.251) (Figs. 2A and 2B). As shown in Figs. 2C and 2D, motility of the parental strain increased from 14.6 mm±0.28 in the absence of NE to 18.8 mm±0.3 (*p* = <0.0001) in the presence of NE. Similarly, motility of the *qseC* mutant increased from 14.10 mm±0.31 without NE to 19.0 mm±0.56 with NE (*p* = <0.0001) and motility of the *qseC-*complemented mutant also increased from 10.0 mm±0.6 in the absence of NE to 17.0 mm±0.5 (*p* = <0.0001) in response to NE (Figs. 2C and 2D).

### Regulatory effects of Hha on the expression of motility are of greater magnitude than QseBC

**Figure 3:**
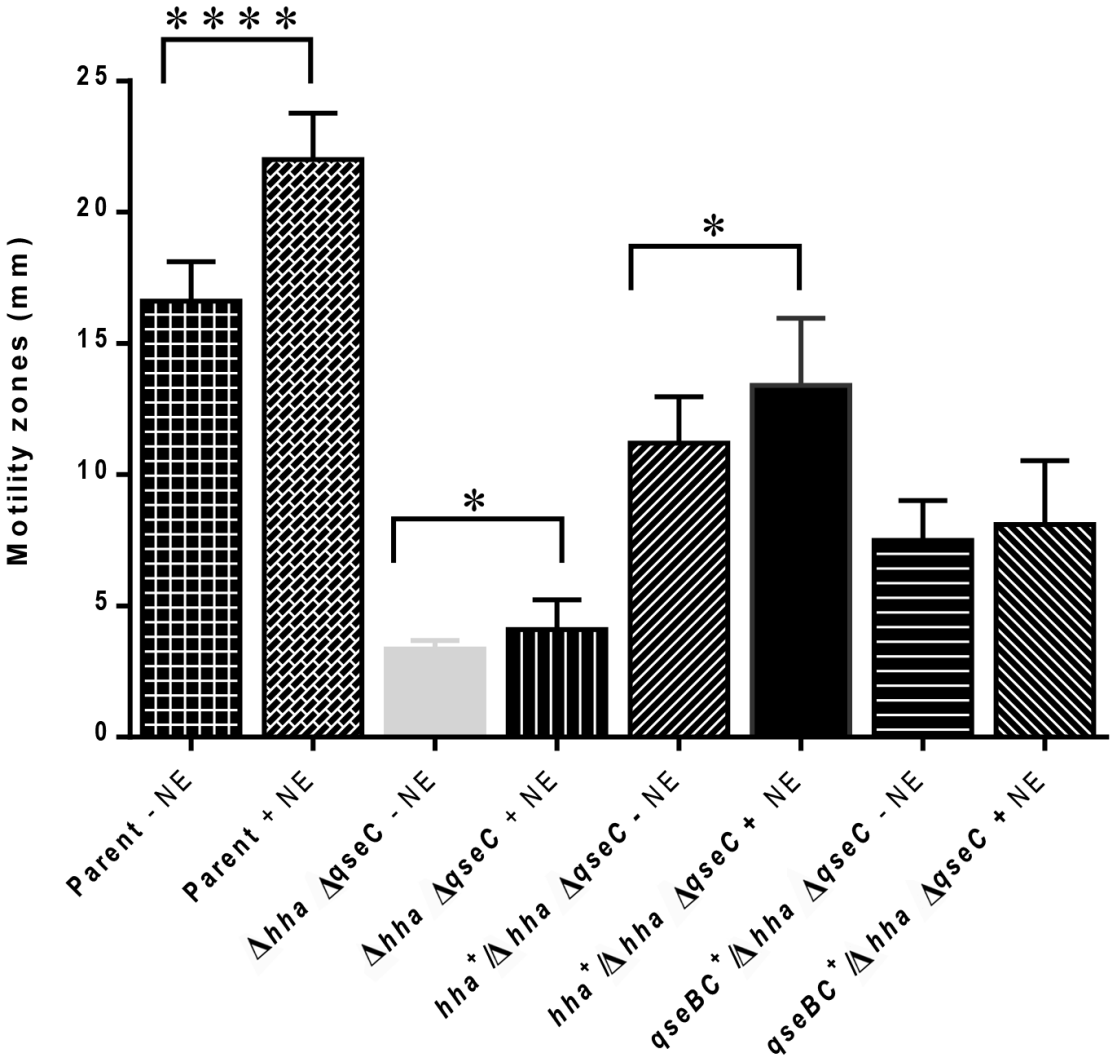
Relative contributions of *hha* and *qseC* to the regulation of motility. Relative motility of a *hha qseC* double deletion mutant was compared to the same mutant complemented with a plasmid-cloned copy of *hha* or *qseC* on the soft-agar motility plates lacking or supplemented with norepinephrine (NE). The motility zones produced after an overnight incubation at 37°C are shown as bar graphs. The bars represent mean diameters of the motility zones (mm) ± SEM computed from the three independent bacterial cultures of each strain. Significance of the difference in motility of a strain grown on a soft-agar motility medium lacking or containing NE is shown by asterisks above the brackets. * (*p* = 0.05); **** (*p = *<0.0001).

The results using single gene deletions, as described in Figs. 1 and 2, provided the first line of evidence that Hha affects motility by a greater magnitude than QseBC. In order to confirm these results, we examined the motility phenotypes expressed by the *hha qseC* double deletion mutant (NADC 6528) (Table 1) with or without complementation with *hha-* or *qseBC*-recombinant plasmids and in the presence or absence of NE. The motility of the *hha qseC* mutant was highly and significantly compromised (3.0 mm±0.11 without NE and 4.0 mm±0.36 with NE; *p* = <0.0001) when compared to the motility phenotypes expressed by the parental strain (17.0 mm±0.48 without and 22.0±0.56 with NE) (Fig. 3), *hha* (9.0 mm±0.47 without and 12.0 mm±0.33 with NE) (Figs. 2A and 2B) and *qseC* mutants (14.0 mm±0.31 without and 19.0±0.56) (Figs. 2C and 2D). *In trans* complementation of the *hha qseC* double deletion mutant with *hha*-recombinant plasmid increased the motility of this mutant to 11.0 mm±0.56 without NE and to 13. 4 mm±0.81 with NE (*p* = 0.0492) (Fig. 3). Overall, we observed about 70% increased motility of *hha-*complemented *hha qseC* mutant (11.0 mm±0.56; *p* = <0.0001) compared to the non-complemented *hha qseC* mutant (3.30 mm±0.11) in the absence of NE. In the presence of NE, there was also about 70% increase in the motility of *hha-*complemented *hha qseC* deletion mutant (13.4 mm±0.81, *p* = <0.0001) compared to the non-complemented *hha qseC* mutant (4.0 mm±0.36). On the other hand, complementation with *qseBC*-carrying plasmid (pSM639) increased motility of the *hha qseC* mutant by a smaller magnitude than complementation of this mutant with the *hha-*carrying plasmid (Fig. 3). For example, as shown in Fig. 3, complementation with the *qseBC-*carrying plasmid restored only 55% (7.5 mm±0.48 without NE) and 49% (8.0 mm±0.77 with NE) of the parental motility to the *hha qseC* mutant compared to 70% of the parental motility restored to the *hha qseC* mutant upon complementation with the *hha-*carrying plasmid in the absence (11.0 mm±0.56; *p* = <0.0001) or presence (13.4 mm±0.81; *p* = 0.0001) of NE. Thus, the data presented in Fig. 3 indicates that *hha* exerts greater control than *qseBC* in regulating motility of EHEC O157:H7.

### Hha is hierarchically superior to QseBC in regulating motility of EHEC O157:H7

**Figure 4:**
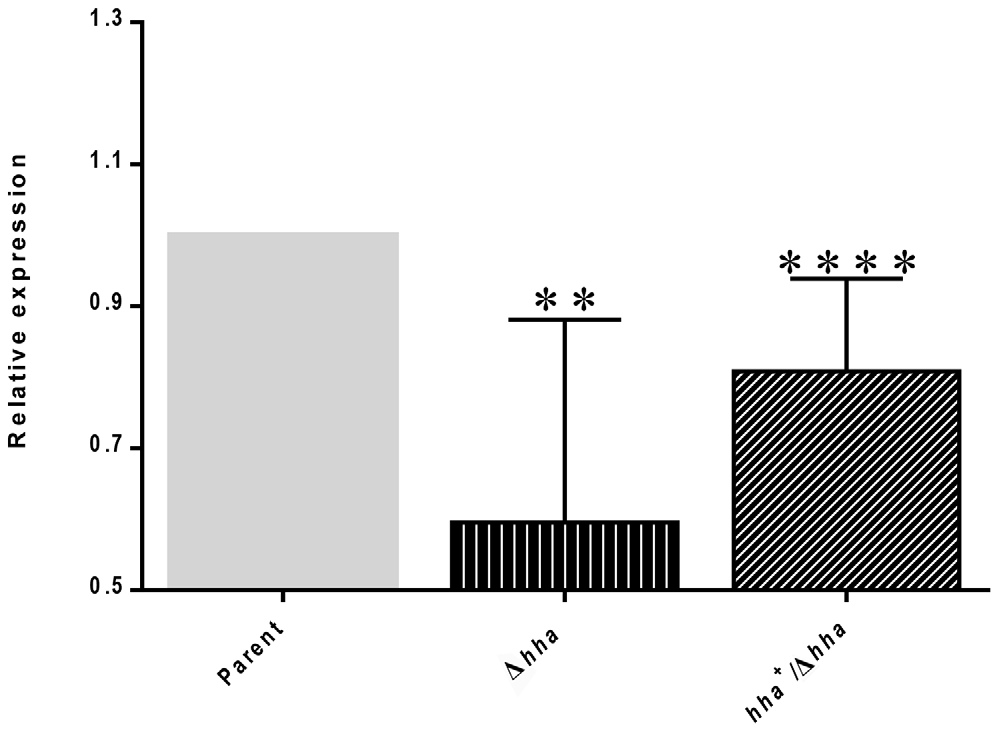
Transcriptional activation of *qseC* by Hha. The effect of *hha* on the transcription of *qseC* was determined by a QRT-PCR assay using a DNA-free RNA prepared from bacterial strains grown in DMEM for 5 h to an A_600_ of 1.1 to 1.2. The relative expression of *qseC* in the parent, *hha* (Δ*hha*), and *hha*-complemented (*hha^+^/*Δ*hha*) mutant strains is shown in the bar graph as means ± SEM of three independent bacterial cultures and nine technical replicates. Significant (*p*<0.05) differences in *qseC* transcription between the parent and mutant strains were determined using Mann-Whitney test. ** (*p* = 0.0019); **** (*p* = <0.0001).

Since complementation with the *hha-*carrying plasmid (pSM197R) restored highest magnitude of motility to the *hha qseC* mutant compared to that restored by complementation of this mutant by the *qseBC-*carrying plasmid, we wanted to determine if the reduced expression of *qseBC* in the *hha* mutant strain was responsible for this disparity. QRT-PCR analysis of the total, DNA-free RNA showed 50% reduction in the transcriptional level of *qseC* in the *hha* deletion mutant (0.6±0.1; *p* = 0.0019) compared to the parental strain (1.00) (Fig. 4). The complementation of the *hha* mutant with the *hha*-carrying plasmid restored transcriptional levels of *qseC* to about 80% (0.81±0.04) of that in the parental strain (1.00) (Fig. 4) indicating that *hha* might be regulating the transcription of the *qseBC* genes.

## Discussion

The expression of flagellar motility in *E. coli* including EHEC O157:H7 requires at least 50 flagellar genes divided into three classes based on the temporal order of their transcription [34]. The *flhDC* genes of class I flagellar operon encode the master regulator FlhDC, which is essential for the expression of all other flagellar genes [49]. Hha, a nucleoid-associated protein [50], and QseBC, encoding a two-component signal transduction system, are among the multitude of transcriptional systems that activate motility in EHEC O157:H7 by activating *flhDC* expression [14], [41], [44]. Here we have shown that Hha exerted greater regulatory effect on motility compared to QseBC as motility of the *hha* deletion mutant was severely compromised compared to that of the parental and the *qseC* deletion mutant strains.

The use of single gene deletion mutants lacking either *hha* or *qseC* showed that Hha is a major activator of motility of EHEC O157:H7 compared to QseBC. By using a *hha qseC* double deletion mutant we were able to demonstrate that the double mutant suffered the highest reduction in motility confirming that both Hha and QseBC contribute to parental-type motility in EHEC O157:H7. Furthermore, complementation of the *hha qseC* mutant with a *hha*- or *qseBC-*carrying plasmid provided data about the relative contributions of Hha and QseBC to the regulation of motility. The complementation analysis confirmed greater regulatory control of Hha on the expression of motility versus that of QseBC. In addition, complementation with *hha* not only restored greater proportion of the parental motility to the *hha qseC* mutant, *hha* complementation also allowed significant increases in bacterial motility in response to norepinephrine. In contrast, the complementation of the *hha qseC* mutant with a *qseBC*-carrying plasmid revealed that the QseBC pathway restored lesser proportion of the parental motility and failed to confer the ability to respond to norepinephrine in the absence of the *hha* gene. Thus, these data indicated that the cumulative regulatory activities of both Hha and QseBC control the expression of the parental motility phenotype in EHEC O157:H7, albeit Hha plays a greater regulatory role in controlling the motility. Although the QseBC encoded signaling system is reportedly essential for the sensing of bacterially produced anuoinducer-3 (AI-3) and mammalian stress hormones epinephrine and norepinephrine in order to enhance motility [14], [44], [48], both *hha* and *qseC* deletion mutants showed significant increases in their motilities in response to norepinephrine. The abilities of the *hha* and *qseC* deletion mutants to respond to norepinephrine are similar to those reported for the *qseC* deletion mutants of *Salmonella enterica* serovar Typhimurium [51]. Collectively, these findings suggest that other yet unknown genetic systems could allow the *hha* as well as the *qseC* mutants to respond to norepinephrine. It is also possible that more than 30 response regulators identified in *E. coli* could, through a cross-talk with a sensory kinase of a specific two-component system [44], [52], induce the expression of other unknown gene(s) enabling the *hha* and *qseC* mutants to respond to norepinephrine and up-regulate their motility.

Hha is a global regulator that specifically controls the expression of horizontally acquired genes, many of which are present as pathogenicity islands in *E. coli* and other members of *Enterobacteriaceae* [32], [35], [50], [53], [54]. The Hha, a nucleoid-associated protein, reportedly interacts with H-NS and other proteins of the Hha/YmoA family to control gene expression through structuring of DNA and modulating its topology in response to osmolarity and temperature [50], [55]. However, we and others have shown that Hha also interacts directly with the promoter elements of transcriptional regulators that in turn control the expression of virulence genes, flagellar motility, and curli fimbriae in EHEC O157:H7 and *Salmonella enterica* serovar Typhimurium [32], [35], [53]. Since complementation with a *qseBC*-carrying plasmid restored the parental level motility to the *qseC* but not to the *hha qseC* double deletion mutant suggested that *hha* might directly control the expression of the *qseBC* genes. Assessment of the expression of the *qseC* gene in the *hha* deletion mutant confirmed that *qseC* transcription was repressed in the *hha* mutant and complementation with a *hha-*carrying plasmid enhanced *qseC* expression.

In conclusion, we have shown that the expression of the parental motility phenotype of EHEC O157:H7 is collectively and positively controlled by both the *hha* and *qseBC* genes, and increased motility in response to norepinephrine occurs independently of *hha* and *qseBC*. In addition, we also determined that *hha* not only plays a greater regulatory role than *qseBC* in the expression of flagellar motility but it also regulates transcription of the *qseBC* genes making *hha* hierarchically superior over *qseBC* in controlling flagellar motility in EHEC O157:H7.
